# Comparative Proteomics Analysis Suggests that Placental Mitochondria are Involved in the Development of Pre-Eclampsia

**DOI:** 10.1371/journal.pone.0064351

**Published:** 2013-05-09

**Authors:** Zhonghua Shi, Wei Long, Chun Zhao, Xirong Guo, Rong Shen, Hongjuan Ding

**Affiliations:** State Key Laboratory of Reproductive Medicine, Nanjing Maternity and Child Health Care Hospital Affiliated to Nanjing Medical University, Nanjing, China; VU University Medical Center, Netherlands

## Abstract

**Introduction:**

Pre-eclampsia (PE), a severe pregnancy-specific disease characterized by the new onset of hypertension, proteinuria, edema, and a series of other systematic disorders, is a state of widespread mitochondrial dysfunction of the placenta.

**Methods:**

We compared the morphology of mitochondria in pre-eclamptic and normotensive placentae using electron microscopy. To reveal the systematic protein expression changes of placental mitochondria that might explain the pathogenesis of PE, we performed iTRAQ analysis combined with liquid chromatography-tandem mass spectrometry (LC-MS/MS) on differentially expressed placental mitochondria proteins from 4 normotensive and 4 pre-eclamptic pregnancies. Bioinformatics analysis was used to find the relative processes that these differentially expressed proteins were involved in. Three differentially expressed proteins were chosen to confirm by Western blotting and immunohistochemistry.

**Results:**

Morphological data demonstrated degenerative and apoptotic changes in the mitochondria of pre-eclamptic placentae. We found four proteins were upregulated and 22 proteins were downregulated in pre-eclamptic placentae compared with normotensive placentae. Bioinformatics analysis showed that these proteins were involved in many critical processes in the development of pre-eclampsia such as apoptosis, fatty acid oxidation, the respiratory chain, reactive oxygen species generation, the tricarboxylic acid cycle and oxidative stress.

**Conclusions:**

This preliminary work provides a better understanding of the proteomic alterations of mitochondria from pre-eclamptic placentae and may aid in our understanding of the importance of mitochondria in the development of pre-eclampsia.

## Introduction

Pre-eclampsia (PE) is a severe pregnancy-specific disease characterized by the new onset of hypertension, proteinuria, edema, and a series of other systematic disorders after 20 weeks' gestation. It is one of the leading causes of maternal and perinatal morbidity and mortality, affecting 5% to 7% of pregnancies [1]–[4]. Treatment is based on clinical features and prevention of complications, with the only intervention that effectively reverses the syndrome being delivery [5].

To date, the etiology and pathogenic mechanisms of PE remains vague, although a growing body of evidence indicates that dysfunction of placental mitochondria is the first step in the pathophysiological cascade leading to pre-eclampsia [5]. Torbergsen [6] indicated a high incidence of pre-eclampsia in a family with mitochondrial dysfunction. Wang and Walsh [7] reported that placental mitochondria are an important source of oxidative stress in PE and that increased mitochondrial lipid peroxidation may cause mitochondrial dysfunction in pre-eclamptic placentae. A series of studies have identified alterations in some placental mitochondria proteins in PE. Shibata [8] identified mitochondrial protein peroxiredoxin III (PRDX3) as a member of the novel antioxidant proteins designated peroxiredoxins in PE. Ekambaram [9] reported that placental mitochondrial HSPA4/HSP70 showed significant overexpression in PE and further implicated HSPA4 in the protection against oxidative stress in PE. Hung [1] found that release of cytochrome C from mitochondria was significantly increased and was associated with increased caspase 3 in PE placentae. The application of mitochondrial proteomics has shed some light on the diagnosis and treatment of many diseases associated with mitochondria. Furthermore, comparison of the mitochondrial proteome from healthy and diseased tissues could aid in the identification of biomarkers for early diagnosis and identification of pathologies associated with mitochondrial dysfunction [10]. Therefore, we hypothesized that investigation of the changes in the mitochondrial placental proteome are required to elucidate the pathophysiological mechanisms of pre-eclampsia.

## Materials and Methods

### Subjects and sample collection

The study design was approved by the Ethical Committee of the Medical Faculty of Nanjing Maternity and Child Health Care Hospital. Patients registered in the department of Obstetrics and Gynecology, Nanjing Maternity and Child Health Care Hospital were enrolled in this study. The informed consents were obtained from these patients. Thirty-five severe pre-eclamptic samples (including 15 preterm preeclampsia cases and 20 term preeclampsia cases) were obtained from primiparous pregnant women (aged 24–31 years) at pregnancies (33–38 weeks) at the time of elective cesarean section. Cases of severe pre-eclampsia (SPE) was classified as patients with adverse outcomes, a BP measurement exceeding 160/110 mmHg on at least two different readings within a 4-h period, proteinuria ≥2 g/day (in 24-h harvest), and/or low platelet counts (<100,000), and/or elevated liver enzymes (alanine transaminase [ALT] >45 U/l, aspartate aminotransferase [AST] >45 U/l, and/or lactate dehydrogenase [LDH] >670 U/l). And Thirty-five controls (15 preterm controls and 20 term controls) were chosen in this study (Table 1). Specific exclusion criteria for the study of both the cases and control groups included diabetes mellitus, renal disease, maternal infection, smoking, chemical dependency, assisted reproductive technology (ART) treatment, multiple pregnancies, fetal congenital anomalies and any other confounding pathology (such as: intrahepatic cholestasis of pregnancy (ICP), hyperthyroidism and hypothyroidism). The indications of cesarean section for control groups at pregnancies (33–39 weeks) were either the presence of breech presentation with premature rupture of membranes or placenta previa with bleeding. Maternal infection of both control and SPE group were excluded by testing blood leukocytes, C-reactive protein, serum procalcitonin and bacteria culture of the cervical secretions. Approximately 1 g of placenta tissue was dissected from the maternal side of the placentae (in the central part, exclusive of calcified area) and rinsed briefly in 0.9% saline, before being snap-frozen in liquid nitrogen. The histopathological characteristics were evaluated after standard preparation of formalin fixed paraffin embedded sections (5 μm thick), stained with hematoxylin and eosin (HE). Patients or their family members were fully informed of the study and signed informed consent forms of their own accord. This study was approved by the Ethics Committee of Nanjing Maternity and Child Health Care Hospital (project No. NJFY-201232).

**Table 1:** Characteristics and outcomes of control and SPE cases.

	*Age (years)*	*Gestational age (weeks)*	*Systolic Blood pressure (mmHg)*	*Diastolic Blood pressure (mmHg)*	*Proteinuria (g/24h)*	*Platelets (×10^9^/L)*	*ALT (U/l)*	*AST (U/l)*	*Birth Weight of infant (g)*	*Placental Weight (g)*
***C(n = 35)***	27.06±2.17	37.01±2.13	119.4±7.1	72.1±6.3	0	249±29	28.5±4.1	34.5±4.7	3507±568	521±152
***P(n = 35)***	27.4±2.34	36.58±1.73	171.6±10.3	105.1±4.2	3.1±0.89	171±18	53.6±5.3	36.2±3.1	2688±320	397±111
***P value (P vs C)***	0.264	0.178	0.000	0.000	0.000	0.001	0.000	0.053	0.023	0.015

Data are presented as mean ± SEM.

C: Control, P: Patient.

### Isolation of placental mitochondria

Isolated placenta tissue (1 mg) was homogenized in 7 ml mitochondrial isolation buffer (10 mM sucrose, 200 mM mannitol, 5 mM HEPES, 1 mM EGTA). The pH was adjusted to 7.4 with KOH. The homogenate was centrifuged at 3,000×*g* for 5 min followed by 6000×*g* for 5 min in the same tube in order to pellet nuclei and cellular debris. The resulting supernatant was decanted to a new tube and centrifuged at 18000×*g* for 20 min to yield a greenish pellet enriched in mitochondria. Transmission electron microscopy (TEM) of mitochondrial homogenates and Western blot using antibodies against marker proteins form different cellular compartments (LDH, GRP78 and COX IV) were performed to evaluate their purity.

### Transmission electron microscopy

Transmission electron microscopy (TEM) of placental mitochondria was performed to evaluate the difference of mitochondria between SPE and control groups. TEM was conducted as previously described [1], [14]. Enriched placental mitochondria from the control and SPE groups were fixed by immersion in 3% glutaraldehyde−0.3% hydrogen peroxide in 0.1 mol/L 1,4-piperazine diethane sulfonic acid (PIPES) buffer (pH 7) for TEM analysis. After fixation at room temperature for 2 h, samples were washed for 30 min in 0.1 mol/L PIPES buffer. Secondary fixation was achieved by immersing the tissue specimen in 1% osmium tetroxide in PIPES buffer for 1 h at room temperature. After three washes in buffer, samples were dehydrated in a graded ethanol series and embedded in Araldite epoxy resin. Ultrathin sections (50 nm) were cut on a Leica Ultracut R Microtome. Sections were counterstained with uranyl acetate, followed by lead citrate, before examination using a Philips H-600 electron microscope (Hitachi).

### Protein preparation and iTRAQ labeling

Briefly, 100 μg mitochondrial proteins from 8 cases (4 SPE cases and 4 controls) were precipitated with cold acetone for 1 hour at −20°C and resuspended in 20 μl dissolution buffer. The information of these 8 patients was shown in Table 2. After protein reduction and alkylation followed by overnight digestion with trypsin, the peptides were labeled with the iTRAQ regents for 1 h at room temperature according to the kit protocol (Applied Biosystems, USA). The iTRAQ regents were used to label the peptides as follows: iTRAQ tag 113, control-1; 114, control-2; 115, control-3; 116, control-4; 117, patient-1; 118, patient-2; 119, patient-3; 121, patient-4 [11]–[13]. The samples were then pooled and fractionated by strong cation exchange (SCX), using a BioLC HPLC column (Dionex, Surrey, UK), and analyzed by LC-MS/MS as previously described [15], [16].

**Table 2:** Characteristics and outcomes of control and SPE cases used in the proteomic analysis.

	*Age (years)*	*Gestational age (weeks)*	*Systolic Blood pressure (mmHg)*	*Diastolic Blood pressure (mmHg)*	*Proteinuria (g/24h)*	*Platelets (×10^9^/L)*	*ALT (U/l)*	*AST (U/l)*	*Birth Weight of infant (g)*	*Placental Weight (g)*
***C1***	30	39.9	126	75	0	268	23.9	35	3788	610
***C2***	25	35.1	116	67	0	268	25.9	28.1	2654	358
***C3***	28	38.1	113	62	0	220	22.1	27.9	3791	560
***C4***	23	39.9	126	73	0	221	22.0	34.9	3793	640
***C***	26.5±3.1	38.3±2.3	120.3±6.7	69.3±5.9	0	244±27	23.5±1.8	31.5±4.0	3067±728	542±110
***P1***	31	37.7	153	102	3.5	183	46.8	41.6	3013	493
***P2***	26	35.4	171	109	4.3	158	50.3	33.2	2250	282
***P3***	29	37.9	153	102	3.1	153	47.5	37.2	2789	567
***P4***	24	37.6	169	101	2.8	182	45.1	39.6	2700	494
***P***	27.5±3.1	37.2±1.1	161.5±9.0	103.5±3.6	3.4±0.65	169±16	47.4±2.2	37.9±3.6	2671±389	459±107
***P value (P vs C)***	0.333	0.240	0.000	0.000	0.000	0.002	0.000	0.030	0.003	0.088

Data are presented as mean ± SEM

C: control, P: Patient

### LC-MS/MS analysis

The nano-LC-MS/MS experiments were performed with LTQ-Orbitrap MS (Thermo Fisher, CA, USA) equipped with a nanoelectrospray ion source. The LTQ-Orbitrap instrument was operated in positive ion mode. An Agilent 1100 series liquid chromatography system equipped with a reverse-phase microcapillary column (0.075×150 mm, Acclaim® PepMap100 C18 column, 3 μm, 100 A; Dionex, Sunnyvale, CA) was used, with the mobile phase consisting of buffer A (0.1% formic acid in H_2_O) and buffer B (0.1% formic acid in ACN). The analytical conditions were set at a linear gradient of buffer B (from 0% to 60% in 60 min) with a flow rate adjusted to 200 nL/min. The column was re-equilibrated at initial conditions for 10 min. For analysis of mitochondria from human placenta, one full MS scan was followed by three MS/MS scans on the three highest peaks.

### Database search and data analysis

The MS/MS spectra acquired from precursor ions were submitted to Mascot http://www.matrixscience.com version 2.2.04 (Matrix Science, London, UK), which was used for protein identification and iTRAQ reporter quantification. Full scan tolerance was 5 ppm, MS/MS tolerance was 0.9 Da, and up to two missed cleavages were accepted. Fixed modifications were those originating from the iTRAQ protocol: iTRAQ-8plex of tyrosine was set as variable modifications. The threshold of significance was set to 0.001, which resulted in a false discovery frequency of less than 0.003 when searched in Mascot against the decoy database of random sequences.

For identification of differentially expressed proteins, the cutoffs for the fold change and P value (student's t-test) were set to 1.5 and 0.05, respectively.

### Pathway analysis by PathwayStudio

An analysis of cellular processes influenced by differentiated mitochondrial proteins in control placentae compared with severe pre-eclampsia placentae was performed using PathwayStudio (Version 7.0) software (Ariadne Genomics, Inc, Rockville, Mass, USA). The text-mining software uses a database of molecular networks that are assembled from scientific abstracts and a manually curated dictionary of synonyms to recognize biological terms. The cellular processes influenced by the various treatments were determined by searching the database for the imported gene/protein and for the cellular processes in which the imported genes/proteins are involved. In our analysis, each identified cellular process was confirmed manually using the relevant PubMed/Medline hyperlinked abstracts.

### Western blot analysis

Samples containing 50 µg of protein were electrophoresed on a 12% SDS polyacrylamide gel and transferred to a nitrocellulose membrane (GE Healthcare, San Francisco, CA, USA). The membranes were blocked in Tris-buffered saline (TBS) containing 5% non-fat milk powder for 1 h and then incubated overnight in polyclonal anti-LDH (1∶200, Abcam, USA), anti-COX IV (1∶100, Abcam, USA), anti- GRP78 (1∶1000, sigma, USA), anti-TFRC (1∶100, Abcam, USA), anti-PRDX3 (1∶1000, Abcam, USA), anti-HSPE1 (1∶500, Abcam, USA) and GAPDH (1∶1000, Kangcheng, China) diluted in TBS/5% non-fat milk powder. GAPDH expression was analyzed as a control. Membranes were washed three times (10 min each) with TBS and then incubated for 1 h with horseradish peroxidase (HRP)-conjugated goat anti-rabbit IgG or goat anti-mouse IgG (1∶1000; Beijing ZhongShan Biotechnology CO., China). Specific proteins were detected using an ECL kit and AlphaImager (FluorChem5500, Alpha Innotech, San Leandro, CA, USA). The protein expression level was analyzed using AlphaEaseFC software (Alpha Innotech, San Leandro, CA, USA).

### Immunohistochemistry

Formalin-fixed tissues were embedded in paraffin, sectioned at 5 μm, and mounted on silane-coated slides. The sections were dewaxed and rehydrated through descending grades of alcohol to distilled water, followed by blocking of endogenous peroxidase using 3% (v/v) hydrogen peroxidase in phosphate buffered saline (PBS). The sections were subjected to microwave antigen retrieval in 0.02 M EDTA, washed in PBS and blocked with goat serum (Beijing ZhongShan Biotechnology,) for 2 h, then incubated overnight at 4°C with polyclonal anti-TFRC (1∶100, Abcam), anti-PRDX3 (1∶200, Abcam), anti-HSPE1 (1∶100, Abcam). Following three washes in PBS, the sections were incubated with HRP-conjugated secondary antibody (1∶1,000; Beijing ZhongShan Biotechnology) for 1 h at room temperature. Immunoreactivity was demonstrated using diaminobenzidine (Sigma, St. Louis, MO, USA) for increased sensitivity, which produced a brown insoluble precipitate at immunopositive sites. Sections were counterstained with hematoxylin and mounted with a cover glass. The negative controls were incubated with IgG controls. All immunostained sections were evaluated in a blinded manner by two observers.

### Statistics

Statistical differences between groups were evaluated using the Student's *t*-test. One-way analysis of variance (ANOVA) followed by the Tukey's post-hoc test was used to examine differences among multiple groups. *P-*values less than 0.05 were considered to indicate statistical significance.

## Results

### Characteristics of the study population

The maternal age, gestational age, and parity in the control group did not differ significantly from those in women with SPE group. Systolic and diastolic blood pressures were significantly higher in the SPE group than in the control group (*P*<0.05). The amount of protein in a 24-h urine collection for the SPE group was 3.1±0.89 g/24 h, although that for the control group was 0. There were significant differences in the levels of ALT, placental weight and platelet counts, but no differences were detected in AST. Birth weight of the infant was significantly lower in the SPE group than in the control group (Table 1).

### Characterization of isolated mitochondria

**Figure 1:**
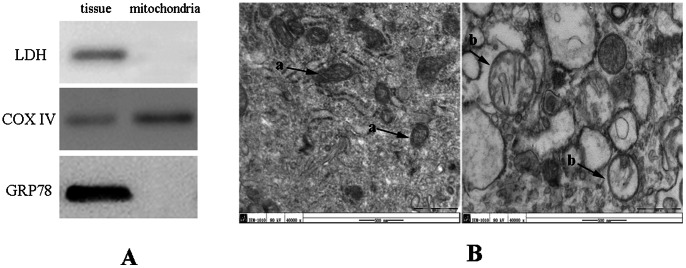
Characterization of isolated mitochondria from placenta. (A) Western blotting images of 3 marker proteins in the whole-tissue protein samples and the isolated mitochondrial protein samples. (B) Electron micrographs of isolated mitochondria from control (left) and SPE placenta (right). Images of isolated mitochondria from control placentae showed a clearly defined electron-dense matrix with particles (respiratory enzyme elements) and intact mitochondrial membranes (a). Some degenerative changes and swollen mitochondria were observed in SPE placentae (b).

The purity of isolated mitochondria was assessed by Western blotting and electron microscopic observations (TEM). Figure 1A showed Western blotting images of extracted from isolated mitochondrial samples against known marker proteins from cytoplasm (LDH), mitochondria (COX IV) and endoplasmic reticulum (GRP78). Mitochondrial protein COX IV was specifically detected in isolated mitochondrial sample and this sample lacked any detectable contamination of abundant cytosolic (LDH) and endoplasmic reticulum proteins (GRP78). Figure 1B showed the high-magnification images of mitochondria from normotensive placentae and PE placentae. The left image showed a clearly defined electron-dense matrix with particles (respiratory enzyme elements) and intact mitochondrial membranes in normotensive placentae. However, the appearance of the mitochondria in the PE placentae showed in the right image confirmed a state of intermittent anoxia. Some degenerative changes and swollen mitochondria were observed. In contrast to the well-defined mitochondrial matrix with clear particles observed in the controls, the inner mitochondria in patients with PE had an undefined internal structure without clearly defined particles (Fig. 1B).

### Proteomic analysis of differential expression

**Figure 2:**
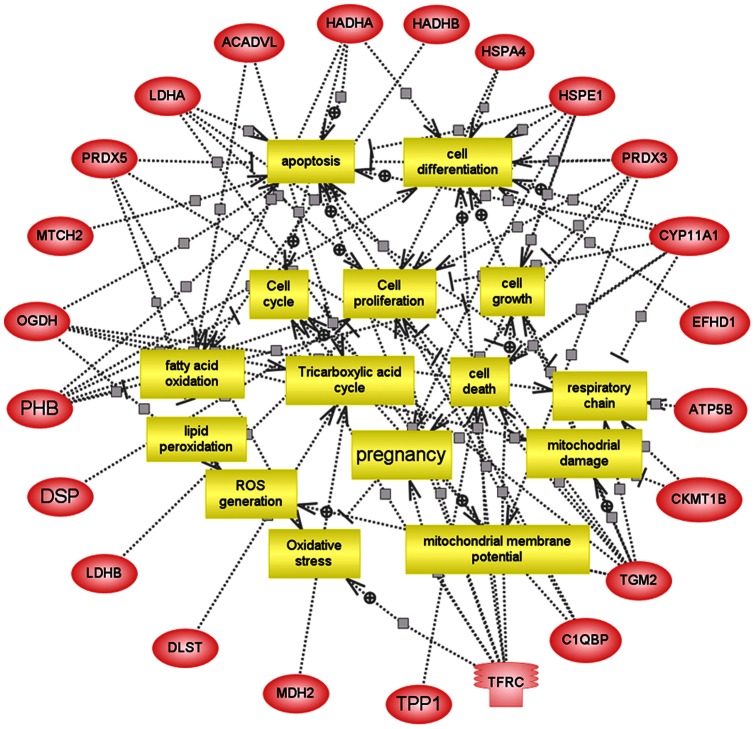
Regulation pathways involving mitochondrial proteins in pre-eclamptic placentae as predicted by PathwayStudio^TM^ software. Proteins are shown as red ovals, regulated processes are represented by yellow squares. Regulation events are displayed with arrows and documented by literature citations.

Comparative proteomic analysis of mitochondrial proteins was performed on 4 normotensive and 4 SPE placentae (Table 2). A total of 26 differentially expressed mitochondrial proteins were identified in comparison between the control and SPE groups (Table 3). To gain a better understanding of the 26 proteins identified in this study, a detailed analysis of cellular processed influenced by these proteins was performed using PathwayStudio^TM^ software. We listed all the differentially expressed mitochondrial proteins identified in the SPE placentae involved in processes such as apoptosis, fatty acid oxidation, the respiratory chain, reactive oxygen species (ROS) generation, the tricarboxylic acid cycle and oxidative stress. The detailed results are shown in Figure 2.

**Table 3:** Full list of the 26 proteins identified by iTRAQ labeling-based proteomics.

Score	%Cov	Accession number	Protein name	Control/SPE	#valid peptide sequences
37.45	45.6	P06576	ATP synthase, H+ transporting, mitochondrial F1 complex, beta polypeptide (ATP5B)	2.01	26
8.03	15.9	Q9BUP0	EF-hand domain family, member D1(EFHD1)	0.25	4
39.7	39.2	P49748	acyl-Coenzyme A dehydrogenase, very long chain (ACADVL)	8.14	21
97.71	32.7	Q00610	clathrin, heavy chain (Hc)	1.75	56
2.45	5	Q07021	complement component 1, q subcomponent binding protein(C1QBP)	5.75	1
14.43	20.9	P12532	creatine kinase, mitochondrial 1A; creatine kinase, mitochondrial 1B(CKMT1B)	7.44	8
39.85	40.1	P05108	cytochrome P450, family 11, subfamily A, polypeptide 1(CYP11A1)	0.29	25
25.59	5.2	P15924	Desmoplakin(DSP)	0.33	13
10.11	10.2	P36957	dihydrolipoamide S-succinyltransferase (DLST)	8.95	5
2.7	8.9	Q9Y2Q3	glutathione S-transferase kappa 1	1.78	2
2.25	6.9	P61604	**heat shock 10kDa protein 1 (chaperonin 10)(HSPE1)**	5.86	1
41.65	33.4	P40939	hydroxyacyl-Coenzyme A dehydrogenase alpha subunit (HADHA)	5.01	22
22.87	29.8	P55084	hydroxyacyl-Coenzyme A dehydrogenase beta subunit(HADHB)	3.07	11
16.05	27.8	P40926	malate dehydrogenase 2, NAD (mitochondrial) (MDH2)	13.55	8
3.26	5.3	Q9Y6C9	mitochondrial carrier homolog 2(MTCH2)	0.49	2
19.86	9.4	Q02218	oxoglutarate (alpha-ketoglutarate) dehydrogenase (OGDH)	2.11	9
6.21	13.3	P30048	**peroxiredoxin 3(PRDX3)**	5.01	4
4.07	12.2	P30044	peroxiredoxin 5 (PRDX5)	1.68	2
16.67	35.7	P35232	Prohibitin(PHB)	1.57	9
62.07	45.7	P02786	**transferrin receptor (p90, CD71)(TFRC)**	3.9	38
26.91	25.8	P21980	transglutaminase 2(TGM2)	4.78	15
9.47	15.3	O14773	tripeptidyl peptidase I(TPP1)	1.59	6
11.17	12.4	P22695	ubiquinol-cytochrome c reductase core protein II	5.39	5
4.98	10.5	P00338	lactate dehydrogenase A(LDHA)	1.51	3
3.8	10.5	P07195	lactate dehydrogenase B(LDHB)	1.60	3
23.01	18.4	P11142	heat shock 70kDa protein 8 (HSPA4)	8.3	13

The key proteins verified by Western blot analysis are highlighted in bold. The corresponding average ratios between the two groups (control/SPE) are given.

### Western blot and immunohistochemical analysis of protein identity

**Figure 3:**
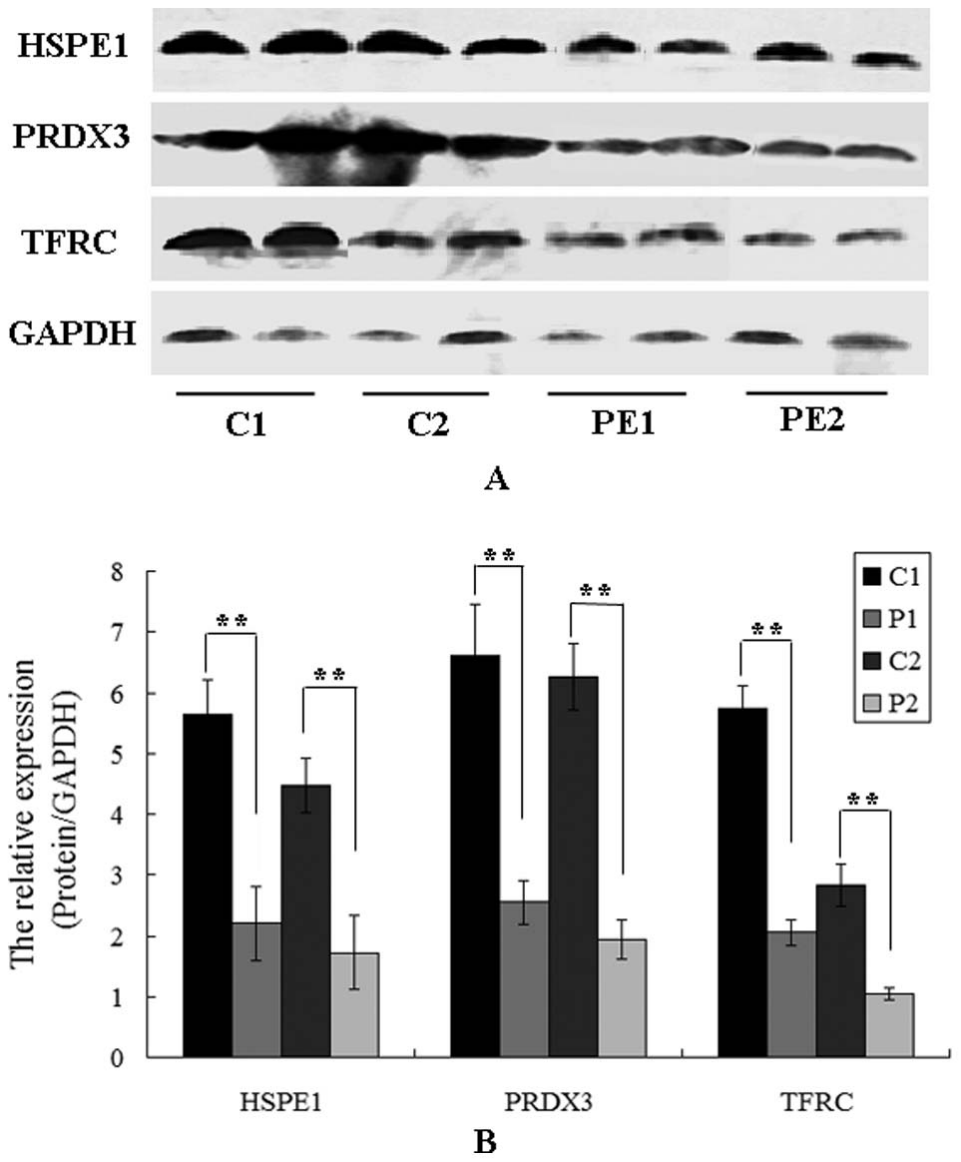
(A) Western blot analysis with anti-HSPE1, anti-PRDX3, anti-TFRC and GAPDH polyclonal antibodies was performed on aliquots of total protein extracts prepared from mitochondrial proteins from 35 SPE cases and 35 controls(C1: term control; C2: preterm control; P1: term SPE cases; P2: preterm SPE cases). (B) Comparison of protein expression levels of HSPE1, PRDX3 and TFRC in SPE and control groups (C1 vs P1 and C2 vs P2; **, p<0.01). The Y-axis represents the relative quantification of target proteins normalized to GAPDH.

**Figure 4:**
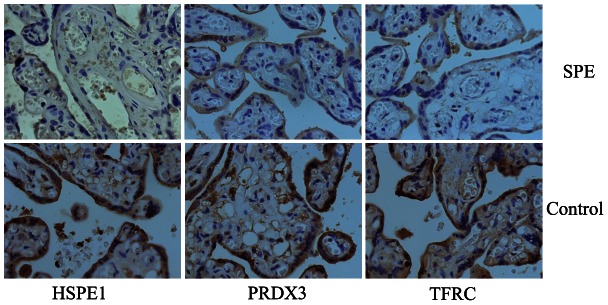
Immunohistochemical staining for HSPE1, PRDX3 and TFRC in the placental tissue of SPE patients and controls. The staining for HSPE1, PRDX3 and TFRC in the cytoplasm of syncytiotrophoblastic and/or cytotrophoblastic cells was more intense in the placental tissue from SPE patients compared with the controls.

Western blot analysis was conducted to validate the LC/MS data. Based on the availability of antibodies, we first analyzed TFRC, PRDX3 and HSPE1 three proteins that were differentially expressed at lower levels in the same samples used for proteome analysis. GAPDH was used as an internal control. The results confirmed the differential protein expression observed by LC/MS. Then we further compared their expression level in all the 15 preterm preeclampsia cases and 20 term preeclampsia cases with that in the preterm and term controls. The result showed that three proteins were all expressed at lower levels in the PE group than in control group (P<0.01, Figure 3) and the levels of the three proteins were all lower in the preterm PE group than in the term PE group (P<0.01). The results also showed that the levels of HSPE1 and PRDX3 were higher in the term controls than in the preterm controls (P<0.01). We also performed immunohistochemical analysis to define the cellular location of these proteins in human placenta. As shown in Fig. 4, all three proteins were expressed in the cytoplasm of the syncytiotrophoblastic and/or cytotrophoblastic cells of human placental tissue. The expression patterns of the three proteins were consistent with the MS and Western blotting analysis.

## Discussion

Pre-eclampsia (PE) is a severe pregnancy-specific disease characterized by the new onset of hypertension, proteinuria, edema, and a series of other systematic disorders. The etiology of PE remains to be elucidated, but a growing body of evidence suggests that a mitochondrial defect underlies the impairment of differentiation and invasion of the trophoblast that leads to this disorder [5]. Several mitochondrial proteins have been shown to be involved in PE, such as peroxiredoxin III (PRDX3) [8], HSP70/HSPA4 [9], Cytochrome C [1] and TFRC [17]. However, the changes in total mitochondrial protein expression in the human pre-eclamptic placenta remain to be systematically analyzed.

The emergence of comparative proteomics enables us to investigate the mitochondrial proteome in a more comprehensive and effective manner, providing a better understanding of the pathogenesis of PE. In this study, we analyzed the expression levels of the major motochondrial proteins extracted from 4 normotensive and 4 SPE placentae and successfully identified 30 differentially expressed proteins using an iTRAQ approach. In an effort to exclude the effects of contamination by other cellular organelles such as the endoplasmic reticulum incurred during the mitochondrial extraction process, all the identified proteins were further analyzed using the GeneOntology and MitoCarta-human databases. A total of 26 of the 30 identified were validated mitochondrial proteins and four originated from the endoplasmic reticulum. Of the 26 mitochondrial proteins, expression of 22 was downregulated in the SPE group compared with that in the normotensive group, while the remaining four were upregulated. The expression patterns of three proteins were analyzed by Western blot and immunohistochemical analysis, thus confirming the LC-MS/MS data (Fig. 3). Trough large sample verification, we also found that the expression levels of these three proteins were down-regulated either in the preterm PE group or the term PE group compared with controls. This result also indicated that mitochondria might be involved in the development of all kinds of severe pre-eclampsia including early onset SPE and late onset SPE.

To further understand the functions of these 26 differentially expressed mitochondrial proteins involved in SPE, we used the PathwayStudio software, which includes an automated text-mining tool that can generate pathways for well-documented proteins from the entire PubMed database and other public sources [18], [19]. The majority of these 26 proteins were found to be involved in diverse biological processes, such as apoptosis, oxidative stress, reactive oxygen species (ROS) generation, fatty acid oxidation (FAO), mitochondrial damage and the tricarboxylic acid cycle.

In PE, endovascular invasion of cytotrophoblasts remains superficial, and uterine blood vessels do not undergo adequate vascular transformation, with consequent placental hypoxia and insufficiency [20]–[24]. And impaired mitochondrial function with reduction of respiratory chain (RC) enzymes and FAO enzymes resulting in abnormal energy production could play a key role in dysfunction of the feto-placental unit [25]. Impaired mitochondria are considered to be one of the major sources of ROS production within cells. ROS may trigger accumulation of secondary mtDNA mutations, exacerbating mitochondrial respiratory defects and consequently, increasing ROS production and oxidative stress from mitochondria [26]. Increased apoptosis, particularly in the syncytiotrophoblast, has been found in placentas from pregnacies complicated by preeclampsia compared with normal pregnancies [27]. It has been proposed that exaggerated apoptosis can be reproduced in trophoblast in vitro by exposure to hypoxia and reactive oxygen species [28]. Hypoxic placentae in PE are likely to be subject to oxidative stress, with disequilibrium between antioxidant defense and the production of ROS. The increased placental apoptosis may be a primary pathologic event or, alternatively, a secondary effect of altered placental oxygenation in preeclampsia. And Redman and Sargent proposed that oxidative stress might stimulate syncytiotrophoblast apoptosis [29]. Pathway analysis of the differentially expression proteins showed that a number of proteins such as TFRC, PRDX5, OGDH and TGM2 were mapped to two or three processes of apoptosis, oxidative stress, ROS generation and mitochondrial damage. These results indicated that mitochondria which were involved in the regulation of oxidative stress and apoptosis play a critical role in the development of PE.

Differentiation of the early embryonic trophoblast (which forms the placenta) and invasion of the trophoblast into the maternal endometrium is a highly energy-consuming process, requiring the synthesis of a large variety of proteins. However, mitochondria are the site of oxidative phosphorylation, the process that yields ATP from pyruvate [5]. We identified some mitochondrial proteins involved in the tricarboxylic acid cycle and the respiratory chain, such as ATP5B, OGDH, DLST, MDH2 and ACADVL. Our data showed that all these five of these enzymes were all downregulated in pre-eclamptic placentae in the third trimester of pregnancy. Although trophoblastic invasion occurs in early pregnancy, our results only prompt that energy production in the pre-eclamptic placenta is insufficient for invasion of the trophoblast and might be a cause of pre-eclampsia. It should be confirmed by a further in vitro functional study using trophoblast cells.

In this study, fatty acid oxidation (FAO) was shown to be a critical process in the development of pre-eclampsia through pathway analysis. FAO had previously been proposed as an important metabolic pathway involved in support of the function of the placenta [30]–[33]. It has been proposed that decreased placental FAO is a contributing factor to the pathophysiology of pre-eclampsia [34], [35] which is consistent with the results of our study showing that mitochondrial FAO was significantly reduced (by 20%) in the pre-eclampsia group compared to the control group. HADHA, HADHB and ACADVL, the critical acyl-CoA dehydrogenases that catalyze the initial step in the FAO pathway [31], were identified and their expression levels were all reduced in the pre-elcamptic group as previously reported [30]. Our results further confirmed that placental mitochondrial FAO plays an important role in the pathophysiology of pre-eclampsia.

In conclusion, this is the first report that uses quantitative mitochondrial proteomics analysis of the placenta to explore the pathophysiology of severe pre-eclampsia. This study of altered mitochondrial proteomes in SPE indicates that pre-eclampsia is a fundamental disorder of placental mitochondria. Although our study is a preliminary work, our further study is still in progress, which contributes to shedding light on the mitochondrial pathogenesis of preeclampsia.

## References

[1] Hung T, Skepper JN, Charnock-Jones DS, Burton GJ (2002) A potent inducer of apoptotic changes in the human placenta and possible etiological factor in Preeclampsia. Circulation Research 90: 1274–81. 12089065 10.1161/01.res.0000024411.22110.aa

[2] Cunningham FG, Leveno KJ, Bloom SL, Hauth JC (2005) Williams Obstetrics. 22nd Edn. New York: McGraw-Hill. 761–808.

[3] Robert JM (2004) Maternal Fetal Medicine, 5th Edn. W. B. Saunders, Philadelphia. 859–899.

[4] Young NK, Hyoung KK, Mohamad W, Nari K, Won SP, et al (2007) Toward a better understanding of preeclampsia: Comparative proteomic analysis of preeclamptic placentae. Proteomics Clin Appl 1: 1625–36. 21136660 10.1002/prca.200700034

[5] Martin W, Hans S, Manfred GM (1998) Pre-eclampsia: a disorder of placental mitochondria? Mol Med Today 4: 286–91. 9743989 10.1016/s1357-4310(98)01293-3

[6] Torbergsen T, Oian P, Mathiesen E, Borud O (1989) Pre-eclampsia: a mitochondrial disease? Acta Obstet Gynecol Scand 68: 145–8. 2589041 10.3109/00016348909009902

[7] Wang Y, Walsh SW (1998) Placental mitochondria as a source of oxidative stress in pre-eclampsia. Placenta 19: 581–6. 9859861 10.1016/s0143-4004(98)90018-2

[8] Shibata E, Nanri H, Ejima K, Araki M, Fukuda J, et al (2003) Enhancement of mitochondrial oxidative stress and up-regulation of antioxidant protein peroxiredoxin III/SP-22 in the mitochondria of human pre-eclamptic placentae. Placenta 24: 698–705. 12828928 10.1016/s0143-4004(03)00083-3

[9] Padmini E, Lavanya S, Uthra V (2009) Preeclamptic placental stress and over expression of mitochondrial HSP70. Clin Chem Lab Med 47: 1073–80. 19728848 10.1515/CCLM.2009.247

[10] Jiang Y, Wang X (2012) Comparative mitochondrial proteomics: perspective in human diseases. J Hematol Oncol 18 5: 11. 10.1186/1756-8722-5-11 PMC3337254 22424240

[11] Wu WW, Wang G, Baek SJ, Shen RF (2006) Comparative study of three proteomic quantitative methods, DIGE, cICAT, and iTRAQ, using 2D Gelor LC-MALDI TOF/TOF. J Proteome Res 5: 651–8. 16512681 10.1021/pr050405o

[12] Ross PL, Huang YN, Marchese JN, Williamson B, Parker K, et al (2004) Multiplexed protein quantitation in saccharomyces cerevisiae using amine-reactive isobaric tagging reagents. Mol Cell Proteomics 3: 1154–69. 15385600 10.1074/mcp.M400129-MCP200

[13] Sun C, Song C, Ma Z, Xu K, Zhang Y, et al (2011) Periostin identified as potential biomarker of prostate cancer by iTRAQ-proteomics analysis of prostate biopsy. Proteome Sci 9: 22. 21504578 10.1186/1477-5956-9-22 PMC3100237

[14] Watson AL, Skepper JN, Jauniaux E, Burton GJ (1998) Susceptibility of human placental syncytiotrophoblastic mitochondria to oxygen-mediated damage in relation to gestational age. J Clin Endocrinol Metab 83: 1697–705. 9589679 10.1210/jcem.83.5.4830

[15] Glen A, Evans CA, Gan CS, Cross SS, Hamdy FC (2010) Eight-plex iTRAQ analysis of variant metastatic human prostate cancer cells identifies candidate biomarkers of progression: An exploratory study. Prostate 70: 1313–32. 20623638 10.1002/pros.21167

[16] Glen A, Gan CS, Hamdy FC, Eaton CL, Cross SS (2008) iTRAQ-facilitated proteomic analysis of human prostate cancer cells identifies proteins associated with progression. J Proteome Res 7: 897–907. 18232632 10.1021/pr070378x

[17] Khatun R, Wu Y, Kanenishi K, Ueno M, Tanaka S, et al (2003) Immunohistochemical study of transferrin receptor expression in the placenta of preeclampticpregnancy. Placenta 24: 870–6. 13129684 10.1016/s0143-4004(03)00138-3

[18] Nikitin A, Egorov S, Daraselia N (2003) Pathway studio-the analysis and navigation of molecular networks. Bioinformatics 19: 2155–7. 14594725 10.1093/bioinformatics/btg290

[19] Pollard HB, Eidelman O, Jozwik C (2006) De novo biosynthetic profiling of high abundance proteins in cystic fibrosis lung epithelial cells. Mol Cell Proteomics 5: 1628–37. 16829594 10.1074/mcp.M600091-MCP200

[20] Roberts JM, Lain KY (2002) Recent insights into the pathogenesis of pre-eclampsia. Placenta 23: 359–72. 12061851 10.1053/plac.2002.0819

[21] Maynard SE, Min JY, Merchan J, Lim KH, Li J, et al (2003) Excess placental soluble fms-like tyrosine kinase 1 (sFlt1) may contribute to endothelial dysfunction, hypertension, and proteinuria in preeclampsia. J Clin Invest 111: 649–58. 12618519 10.1172/JCI17189 PMC151901

[22] Brosens IA, Robertson WB, Dixon HG (1972) The role of the spiral arteries in the pathogenesis of preeclampsia. Obstet Gynecol Annu 1: 177–91. 4669123

[23] Fisher SJ (2004) The placental problem: linking abnormal cytotrophoblast differentiation to the maternal symptoms of preeclampsia. Reprod Biol Endocrinol 2: 53. 15236649 10.1186/1477-7827-2-53 PMC493282

[24] Huppertz B, Kadyrov M, Kingdom JC (2006) Apoptosis and its role in the trophoblast. Am J Obstet Gynecol 195: 29–39. 16579915 10.1016/j.ajog.2005.07.039

[25] Illsinger S, Janzen N, Sander S, Schmidt KH, Bednarczyk J, et al (2010) Preeclampsia and HELLP syndrome: impaired mitochondrial function in umbilical ensothelial cells. Reprod Sci 17: 219–26. 20065299 10.1177/1933719109351597

[26] Kirkinezos IG, Moraes CT (2001) Reactive oxygen species and mitochondrial diseases. Semin Cell Dev Bio 12: 449–57. 11735379 10.1006/scdb.2001.0282

[27] Leung DN, Smith SC, To KF, Sahota DS, Baker PN (2001) Increased placental apoptosis in pregnancies complicated by preeclampsia. Am J Obstet Gynecol 184: 1249–50. 11349196 10.1067/mob.2001.112906

[28] Sharp AN, Heazell AE, Crocker IP, Mor G (2010) Placental apoptosis in health and disease. Am J Reprod Immunol 64: 59–69. 10.1111/j.1600-0897.2010.00837.x PMC3025811 20367628

[29] Redman CW, Sargent IL (2005) Placental debris, oxiative stress and pre-eclampsia. Science 308: 1592–4. 10985960 10.1053/plac.2000.0560

[30] Bartha JL, Visiedo F, Fernández-Deudero A, Bugatto F, Perdomo G (2012) Decreased mitochondrial fatty acid oxidation in placentas from women with Preeclampsia. Placenta 33: 132–4. 22189170 10.1016/j.placenta.2011.11.027

[31] Shekhawat P, Bennett MJ, Sadovsky Y, Nelson DM, Rakheja D, et al (2003) Human placenta metabolizes fatty acids: implications for fetal fatty acid oxidation disorders and maternal liver diseases. Am J Physiol Endocrinol Metab 284: E1098–105. 12582009 10.1152/ajpendo.00481.2002

[32] Haggarty P, Allstaff S, Hoad G, Ashton J, Abramovich DR (2002) Placental nutrient transfer capacity and fetal growth. Placenta 23: 86–92. 11869095 10.1053/plac.2001.0743

[33] Herrera E, Amusquivar E, Lopez-Soldado I, Ortega H (2006) Maternal lipid metabolism and placental lipid transfer. Horm Res 65 Suppl 359–64. 16612115 10.1159/000091507

[34] Rakheja D, Bennett MJ, Foster BM, Domiati-Saad R, Rogers BB (2002) Evidence for fatty acid oxidation in human placenta, and the relationship of fatty acid oxidation enzyme activities with gestational age. Placenta 23: 447–50. 12061861 10.1053/plac.2002.0808

[35] Shekhawat PS, Matern D, Strauss AW (2005) Fetal fatty acid oxidation disorders, their effect on maternal health and neonatal outcome: impact of expanded newborn screening on their diagnosis and management. Pediatr Res 57: 78R–86R. 10.1203/01.PDR.0000159631.63843.3E PMC3582391 15817498

